# AltitudeOmics: Rapid Hemoglobin Mass Alterations with Early Acclimatization to and De-Acclimatization from 5260 m in Healthy Humans

**DOI:** 10.1371/journal.pone.0108788

**Published:** 2014-10-01

**Authors:** Benjamin J. Ryan, Nadine B. Wachsmuth, Walter F. Schmidt, William C. Byrnes, Colleen G. Julian, Andrew T. Lovering, Andrew W. Subudhi, Robert C. Roach

**Affiliations:** 1 Department of Integrative Physiology, University of Colorado Boulder, Boulder, Colorado, United States of America; 2 Department of Sports Medicine/Sports Physiology, University of Bayreuth, Bayreuth, Germany; 3 Altitude Research Center, Department of Emergency Medicine, University of Colorado Anschutz Medical Campus, Aurora, Colorado, United States of America; 4 Department of Human Physiology, University of Oregon, Eugene, Oregon, United States of America; 5 Department of Biology, University of Colorado Colorado Springs, Colorado Springs, Colorado, United States of America; Georgia Regents University, United States of America

## Abstract

It is classically thought that increases in hemoglobin mass (Hbmass) take several weeks to develop upon ascent to high altitude and are lost gradually following descent. However, the early time course of these erythropoietic adaptations has not been thoroughly investigated and data are lacking at elevations greater than 5000 m, where the hypoxic stimulus is dramatically increased. As part of the AltitudeOmics project, we examined Hbmass in healthy men and women at sea level (SL) and 5260 m following 1, 7, and 16 days of high altitude exposure (ALT1/ALT7/ALT16). Subjects were also studied upon return to 5260 m following descent to 1525 m for either 7 or 21 days. Compared to SL, absolute Hbmass was not different at ALT1 but increased by 3.7±5.8% (mean ± SD; n = 20; p<0.01) at ALT7 and 7.6±6.6% (n = 21; p<0.001) at ALT16. Following descent to 1525 m, Hbmass was reduced compared to ALT16 (−6.0±3.7%; n = 20; p = 0.001) and not different compared to SL, with no difference in the loss in Hbmass between groups that descended for 7 (−6.3±3.0%; n = 13) versus 21 days (−5.7±5.0; n = 7). The loss in Hbmass following 7 days at 1525 m was correlated with an increase in serum ferritin (r = −0.64; n = 13; p<0.05), suggesting increased red blood cell destruction. Our novel findings demonstrate that Hbmass increases within 7 days of ascent to 5260 m but that the altitude-induced Hbmass adaptation is lost within 7 days of descent to 1525 m. The rapid time course of these adaptations contrasts with the classical dogma, suggesting the need to further examine mechanisms responsible for Hbmass adaptations in response to severe hypoxia.

## Introduction

Precise regulation of erythropoiesis is critical, as both anemia and excessive polycythemia have detrimental effects on physiological function. Hypoxia is a potent stimulator of erythropoiesis and erythropoietin (EPO) increases within hours of hypobaric hypoxia [1]. However, it is classically thought that elevations in total hemoglobin mass (Hbmass) and red cell volume (RCV) during high altitude acclimatization require several weeks to occur [2], [3]. This delayed increase fits with patterns observed with exogenous EPO administration in healthy humans, where Hbmass/RCV have been consistently reported to remain unchanged within the first 12 days of treatment [4], [5], [6]. Although previous studies examining erythropoeitic adaptations in lowlanders adapting to altitudes greater than 4000 m for periods longer than 4 weeks have consistently reported increases in Hbmass/RCV [7], [8], the time course of erythropoietic adaptation during early (i.e., first 1–3 weeks) high altitude acclimatization is less clear. For example, whereas some studies have found unchanged RCV following 2–3 weeks at 4300 m [9], [10], others have found moderate increases at this same elevation over a similar time course [11], [12]. More recently, studies examining the early time course of changes in Hbmass at lower altitudes (2000 m–3600 m) have reported small (2–3%) but significant increases in Hbmass following 11–13 days [13], [14], [15]. Data from these studies conflict with the classical dogma that at least 3–4 weeks are required for increases in Hbmass to be observed, but this remains a matter of considerable debate [16], [17]. Further investigation of early erythropoietic adaptations to high altitude is warranted and, importantly, data are lacking at elevations greater than 5000 m where the hypoxic stimulus for erythropoiesis is dramatically increased [18], [19].

Gains in Hbmass/RCV obtained during high altitude acclimatization are eventually lost following descent to low altitude, but the time course of this de-acclimatization also remains unclear. Based upon the traditional kinetics of red blood cell production and destruction, large reductions in Hbmass/RCV are expected to take multiple weeks to occur. Recent altitude training studies conducted at elevations between 2000 m–3600 m have reported full or partial retention of altitude-induced gains in Hbmass for 2–3 weeks following descent to sea level [14], [15], [17], with Hbmass eventually returning to baseline sea level values [14], [17]. Hbmass/RCV have also been reported to remain elevated for several weeks following cessation of EPO treatment [5], [6], [20]. These studies suggest that elevations in Hbmass induced with short-term environmental or pharmacologic perturbation decay gradually over several weeks. In contrast, a study of polycythemic high altitude natives from 4380 m reported a rapid loss in RCV of ∼7% within the first week of descent to sea level; this rapid loss in RCV was coupled with increases in several markers of red blood cell destruction [21]. Although this study suggested that an increased rate of red blood cell destruction may cause a rapid reduction in Hbmass following high altitude descent, the high altitude natives studied were severely polycythemic and therefore these results may not extend to subjects with less marked polycythemia. As with studies examining changes in Hbmass during the early phase of acclimatization to altitudes greater than 5000 m, we are unaware of any studies examining the early time course of loss in Hbmass following descent from altitudes greater than 5000 m.

AltitudeOmics was designed as a large collaborative research project examining early high altitude acclimatization/de-acclimatization in multiple physiological systems [22]. As a result of this overall project design, we had the unique opportunity to examine the early time course of erythropoietic adaptations with ascent to and descent from 5260 m in healthy humans. Because rapid changes in plasma volume (PV) occur within the first days of high altitude ascent (i.e., [23], [24], [25], [26], [27] and descent [26], [27], early alterations in hemoglobin concentration ([Hb]) or hematocrit (Hct) do not necessarily reflect changes in Hbmass/RCV. Therefore, we measured Hbmass and blood volume compartments in lowlanders at sea level, on 3 occasions at 5260 m during 16 days of high altitude exposure, and upon initial return to 5260 m following descent to low altitude (1525 m) for either 7 or 21 days.

## Methods

### Study Design

A detailed description of the overall AltitudeOmics study design and subject characteristics is reported elsewhere [22]. The study was approved by the Institutional Review Boards of the Universities of Colorado and Oregon, and by the Human Research Protection Office of the U.S. Department of Defense. Subjects were informed about the possible risks and discomforts involved before giving written and verbal consent to participate.

The data reported here are novel with the exception of the basic characteristics of the 21 AltitudeOmics subjects (12 males, 9 females; age: 21±1 years; height: 176±8 cm; body mass: 70±9 kg) that have been reported previously [22]. Subjects were studied near sea level (130 m; Eugene, OR, USA) and on 4 occasions at 5260 m (Mt. Chacaltaya, Bolivia). Hbmass/BV compartments were measured in duplicate at sea level (SL) during baseline testing, with the mean of 2 tests used as the SL value. Nine to ten weeks after SL testing, subjects were flown via commercial aircraft to El Alto, Bolivia (4050 m) and then immediately driven to 1525 m (Coroico, Bolivia), where they stayed for 2 days. Subjects were then driven to 5260 m on ALT1 and Hbmass/BV parameters were assessed after 9–13 hours at this elevation. Subjects spent days ALT2-ALT4 at 3800 m (La Paz, Bolivia), with a short visit to 5260 m on ALT4, before returning to 5260 m on ALT5. Subjects remained at 5260 m from ALT5 to ALT17, and Hbmass and BV compartments were assessed on ALT7 and ALT16. On ALT17, subjects descended to 1525 m for either 7 (POST7; n = 14) or 21 days (POST21; n = 7). Subjects were transported back to 5260 m on POST7/POST21 and Hbmass/BV measurements were taken following 9–13 hours at this elevation.

Serum ferritin was assessed in all subjects 2–3 weeks prior to baseline testing. All male subjects had initial ferritin levels greater than 20 ng mL^−1^ and none received iron supplementation during the study. Women with initial ferritin levels less than 20 ng mL^−1^ (n = 7) were directed to take oral iron supplements (325 mg ferrous sulfate, 2–3 times daily) for 2–3 weeks prior to baseline testing and until departure to high altitude. One subject ceased supplementation prior to departure to high altitude due to gastrointestinal complaints. No subjects received iron supplementation following departure from SL. The decision not to provide iron supplementation during high altitude acclimatization/de-acclimatization was made based on potential confounding influences [28], [29] of iron supplementation on other physiological responses that were assessed as part of the overall AltitudeOmics project.

Subjects participated in many studies as part of the AltitudeOmics project and some involved blood sampling. At SL, Hbmass/BV assessments were performed prior to any other blood sampling. At high altitude, Hbmass/BV parameters were measured following other blood sampling. The estimated volume of blood withdrawn for sampling at each altitude time point was as follows–ALT1: 212±81 mL; ALT7: 64±26 mL; ALT16: 191±10 mL; POST7/21: 147±46 mL. To examine the effect of blood sampling on Hbmass measured at ALT1, we compared our measured Hbmass values at ALT1 and SL and found that the mean values were not significantly different (see Results). Additionally, when we examined Hbmass across all time points using measured or adjusted (for estimated Hbmass withdrawn due to blood sampling), the statistical significance of our findings remained unaltered. Therefore, we have chosen to report the measured Hbmass values without adjusting for blood withdrawn for sampling but address the magnitude of Hbmass lost due to sampling in the discussion.

### Analytical Methods

#### Hbmass and BV Parameters

Hbmass was measured using the optimized carbon monoxide (CO) rebreathing method [30], [31] with minor modifications. Following at least 20 minutes of seated rest, a venous (v) blood sample (∼2 mL) was obtained from an antecubital vein and used for determination of v[Hb] (OSM3 hemoximeter, Radiometer, Denmark) and vHct (microcentrifugation). The OSM3 was calibrated for [Hb] at regular intervals according to the manufacturers' recommendations. v[Hb] and vHct were analyzed in triplicate. Arterialized capillary blood samples (200 µL) were obtained from a hyperemic earlobe and measured for baseline carboxyhemoglobin (HbCO%) in sextuplicate on the OSM3. End-tidal [CO] was measured using a portable CO detector (Draeger Pac 7000, Draeger, Germany). Subsequently, a bolus of 99.9% CO was administered to subjects from a calibrated syringe into a custom-built spirometer (Spico-CO Respirations-Applikator, Blood Tec, Germany) and rebreathed for 2 minutes along with 3 to 5 L of 100% oxygen.

The volume of CO administered to subjects was chosen to induce a ∼5–6% increase in HbCO%. The volume of CO administered was increased at high altitude based on the reduced barometric pressure to obtain similar ΔHbCO% in tests at sea level and high altitude. However, the largest volume of CO administered was 135 mL (maximal volume of calibrated syringe). The mean ΔHbCO% following the rebreathing procedure was 5.4±0.8%.

Potential CO leaks from the subject or rebreathing apparatus were monitored throughout the rebreathing procedure using 2 portable CO detectors. Due to the effects of CO leaks on measurement error of Hbmass [32], any test in which a CO leak was detected was excluded (total of 6 tests). End-tidal [CO] was measured 4 minutes following initial CO inhalation. Arterialized capillary blood samples (100 µL) were obtained at minutes 6 and 8 following CO inhalation and analyzed in triplicate, with the mean of minute 6 and 8 taken as post-rebreathing HbCO%. The amount of CO remaining in the spirometer was measured using a calibrated syringe and a portable CO detector (Draeger Pac 7000, Draeger, Germany). All data were compiled and used to calculate Hbmass according to previously published formulas [30], [31].

The altitude in the present study was higher than any previous studies employing the optimized CO rebreathing method. To address a potential issue due to differences in oxygen saturation [33] between sea level and high altitude testing conditions, the following minor modification was made. For tests at sea level, subjects breathed a hyperoxic gas mixture (50.5% O_2_, balance nitrogen, P_I_O_2_≈360) for 10 minutes prior to baseline blood sampling and throughout the rest of the procedures, with the exception of the 2-minute CO rebreathing procedure, where 100% O_2_ was rebreathed along with CO. At high altitude (P_B_ = 408 mmHg), subjects breathed 100% O_2_ (P_I_O_2_≈360) for 10 minutes prior to the baseline blood sampling and throughout the rest of the procedures. Hyperoxia was provided at both sea level and high altitude to establish similarly high oxygen saturation levels during the Hbmass procedure in both environments in order to eliminate the influence of oxygen saturation differences on the analytical determination of HbCO% on the OSM3 hemoximeter [33].

A single OSM3 unit was used for all HbCO% measurements during the study. To determine any potential confounding effects of the international transport on the OSM3 hemoximeter or high altitude *per se* on the measurement of HbCO%, we performed a quality control analysis using in-house arterial blood samples that we had obtained prior to this study at 2 levels of HbCO% (high and low HbCO%). These control samples were run in sextuplicate on 5 days during sea level testing and the mean ΔHbCO% between days was 6.84±0.05%; control samples were kept frozen at −80°C until transport–they remained stored on ice during transport and were analyzed in sextuplicate on 2 days within the first week at high altitude. The mean ΔHbCO% between these days was 6.80±0.07%. Thus, the difference in ΔHbCO% between sea level and high altitude was well within the intra-analyzer variability of ΔHbCO% using OSM3 hemoximeters [34], indicating that neither high altitude nor the international transport of the OSM3 had any confounding effects on the measurement of ΔHbCO% that is critical to Hbmass determination.

Measurement errors of Hbmass and BV parameters were calculated from duplicate baseline measurements (n = 19) according to Hopkins [35]. Measurement error for Hbmass was 1.5% (95%CI: 1.1–2.3%)–with duplicate Hbmass tests within a 1-week period, measurement error reflects primarily analytical error as the biological variation over this time frame has been shown to be minimal [36], [37].

RCV, BV, and PV were derived from Hbmass, v[Hb], and vHct as follows:

1. RCV = Hbmass×MCHC^−1^×100^−1^
2. BV = RCV×100×Hct^−1^×0.91^−1^
3. PV = BV−RCV

Hct was multiplied by 0.96 to account for trapped plasma; the constant of 0.91 was included in the BV calculation to correct for the ratio of body hematocrit to peripheral hematocrit [38]. Measurement errors for RCV, PV, and BV were 2.2% (95%CI: 1.6–3.2%), 4.9% (95%CI: 3.6–7.3%), and 3.4% (95%CI: 2.5–5.0%), respectively. For BV compartments, the measurement error is influenced by analytical error of Hbmass, [Hb], and Hct as well as biological variation in PV and total BV.

#### Serum Ferritin and EPO

Whole blood samples were collected following 30 min of rest from a catheter placed in an antecubital vein [22]. The ALT1 ferritin sample was taken after ∼2 hours at 5260 m whereas the ALT1 EPO sample was taken after ∼10 hours at 5260 m. Samples were drawn into 10 mL syringes and immediately transferred into serum collection vacutainers (BD, Franklin Lakes, NJ, USA). These vacutainers were inverted 5 times and then allowed to sit for 30–60 minutes at room temperature to allow for proper clotting. Tubes were spun for 20 minutes at 800 relative centrifugal force at room temperature. Once separated, serum was stored on ice for 10 minutes before being stored in either a −80°C freezer (Eugene), or in a charged nitrogen vapor shipper (Bolivia). Frozen serum samples were transported in charged nitrogen vapor shippers, and then stored at −80°C until analysis. Serum ferritin was assessed via nephelometry (within-run coefficient of variation (CV): 7%; between-run CV: 5%; Siemens BNII Nephelometer, Erlangen, Germany). Serum EPO was assessed in duplicate using a Quantikine IVD Human Epo ELISA kit (intra-assay CV: 4%; inter-assay CV: 6%; R&D Systems, Minneapolis, MN, USA).

#### Missing Data

As mentioned above, 6 Hbmass tests were excluded due to CO leaks. We also missed Hbmass tests due to logistical difficulties (6 tests) and subject discomfort prior to Hbmass testing (1 test). Some v[Hb] or vHct samples (total of 16 tests) were missed for logistical reasons and difficulties with obtaining or processing venous samples; in the case of missing v[Hb] or vHct, data were also excluded from the analysis of changes in BV parameters. The majority of missing tests for Hbmass and BV occurred at ALT1. Ferritin values were missing from 3 tests and EPO values were missing from 8 tests.

#### Statistics

Statistical analyses were performed using Statistical Package for the Social Sciences (version 20, SPSS Inc., Chicago, IL, USA) and Microsoft Excel 2008 (Redmond, WA, USA). We performed linear mixed model statistical analyses to examine our outcome variables across acclimatization using the Mixed procedure in SPSS. A major advantage of linear mixed model statistical analyses is that missing values do not result in casewise deletion of other longitudinal measurements, as is required with repeated-measures analysis of variance. Time (SL, ALT1, ALT7, ALT16), sex, and a time × sex interaction were included in the linear mixed models as fixed factors. Time comparisons were made with SL as the reference. Separate paired-tests were performed to compare ALT7 and ALT16. No adjustments were made for multiple testing. Due to largely reduced subject numbers at ALT1 for Hbmass/BV parameters, we could not be certain that the missing data at ALT1 met the missing-at-random requirement of the linear mixed model. Therefore, we performed paired t-tests to examine changes between SL and ALT1. To examine differences at POST7/POST21 compared to SL and ALT16, we used linear mixed models with time (SL, ALT16, POST), group (POST7, POST21), and a time × group interaction included as fixed factors. The POST timepoint includes POST7 and POST21 measurements, with differences between the groups descending for 7 versus 21 days assessed by comparing the effect of group. Time comparisons were made with POST as the reference–therefore, data from SL and ALT16 were excluded from these analyses for subjects missing the POST7/POST21 time point. Due to the reduced number of female subjects included at POST21 (n = 2), sex was not included in these models. We performed simple linear regressions to examine relationships between variables. For all analyses, statistical significance was accepted when p≤0.05. Data are presented throughout the paper as mean ± SD unless otherwise noted. A complete list of individual data for Hbmass and serum ferritin is provided in Table S1.

## Results

### Subject characteristics and ferritin status during high altitude acclimatization and de-acclimatization

A detailed description of subject characteristics is presented elsewhere [22] –briefly, body mass was reduced by 2.6±1.6 kg after 16 days of high altitude exposure. Serum ferritin levels are presented in Table 1. Ferritin levels were lower in women compared to men. At ALT1, all men had ferritin above 20 ng mL^−1^, whereas 4 of 8 women had ferritin levels below this value. Ferritin levels decreased from ALT1 to ALT16 in both men (−68±16%) and women (−65±26%) and increased following descent from high altitude in both men (+189±196%) and women (+184±283%).

**Table 1:** Serum ferritin during high altitude acclimatization and de-acclimatization.

		Time				Significant Effects	
		SL	ALT1	ALT16	POST	Sex	Time
Serum Ferritin (ng ml^−1^)						M>W	ALT16 <SL; ALT16<ALT1; ALT16<POST
	**M**	63.2±29.0 (12)	66.8±42.2 (11)	25.8±24.0 (12)	52.3±44.0 (11)		
	**W**	28.9±15.5 (9)	19.7±10.9 (8)	7.5±5.2 (9)	19.6±21.3 (9)		

Data are presented as mean ± SD (ng ml^−1^) with the number of subjects indicated in parentheses. The POST measurement took place upon initial return to 5260 m following descent to 1525 m for 7 days (7W, 7M) or 21 days (2W, 4M) duration. Linear mixed model statistical analyses were performed to examine the effects of sex and time with SL as the reference. Paired t-tests were performed to compare ALT16 with ALT1 and POST. Effects were accepted as significant when p≤0.05.

### Hematological adaptations during 16 days of high altitude acclimatization

EPO increased from a baseline level of 8.3±5.0 IU L^−1^ by 4.9±2.8 fold at ALT1 (n = 16), 8.3±8.7 fold at ALT7 (n = 18), and 2.5±1.6 fold at ALT16 (n = 21; all p<0.05 compared to SL). There were no significant correlations between the increases in EPO at ALT1, ALT7, or ALT16 and changes in Hbmass. Data comparing SL and ALT1 for subjects with Hbmass/BV measurements at both time points are presented separately in Table 2 so that the effect of acute altitude on Hbmass and BV parameters can be distinguished from inter-individual variation. We found a non-significant 11 g loss in absolute Hbmass at ALT1 compared to SL (p = 0.206); there was also a trend for relative Hbmass to be slightly (0.3 g kg^−1^) reduced at ALT1 compared to SL (p = 0.056). A small decrease in Hbmass was expected, as Hbmass was assessed after the required blood sampling for other protocol procedures on ALT1. v[Hb], vHct, and BV compartments were not significantly different at ALT1 compared to SL.

**Table 2:** Hematological parameters at sea level and the first day of exposure to 5260 m.

	SL	ALT1	Percent Change
**Hbmass (g)**	723±175 (6,6)	711±173 (6,6)	−1.6±4.4%
**Rel Hbmass (g kg^−1^)**	10.1±1.4 (6,6)	9.8±1.4 (6,6)	−2.7±4.4%
**v[Hb] (g dL^−1^)**	13.9±1.0 (8,4)	14.3±1.5 (8,4)	2.5±6.7%
**vHct (%)**	42.6±2.6 (8,4)	42.6±4.5 (8,4)	−0.3±6.5%
**BV (ml)**	6224±848 (5,3)	6130±517 (5,3)	−0.8±7.5%
**Rel BV (ml kg^−1^)**	83.2±6.6 (5,3)	81.9±7.3 (5,3)	−1.4±7.0%
**PV (ml)**	3907±416 (5,3)	3866±276 (5,3)	−0.2±11.5%
**Rel PV (ml kg^−1^)**	52.4±4.7 (5,3)	51.9±7.3 (5,3)	−0.9±10.9%
**RCV (ml)**	2317±442 (5,3)	2264±417 (5,3)	−2.1±5.0%
**Rel RCV (ml kg^−1^)**	30.8±3.0 (5,3)	29.9±3.3 (5,3)	−2.6±5.6%

Data are presented as mean ± SD with the number of subjects (M,W) indicated in parentheses. This table only includes data for subjects with measures at both time points so that the effect of acute altitude on Hbmass and BV parameters can be distinguished from the inter-individual variation. Paired t-tests were performed for each parameter and none of the differences were statistically significant (all p>0.05).

**Figure 1:**
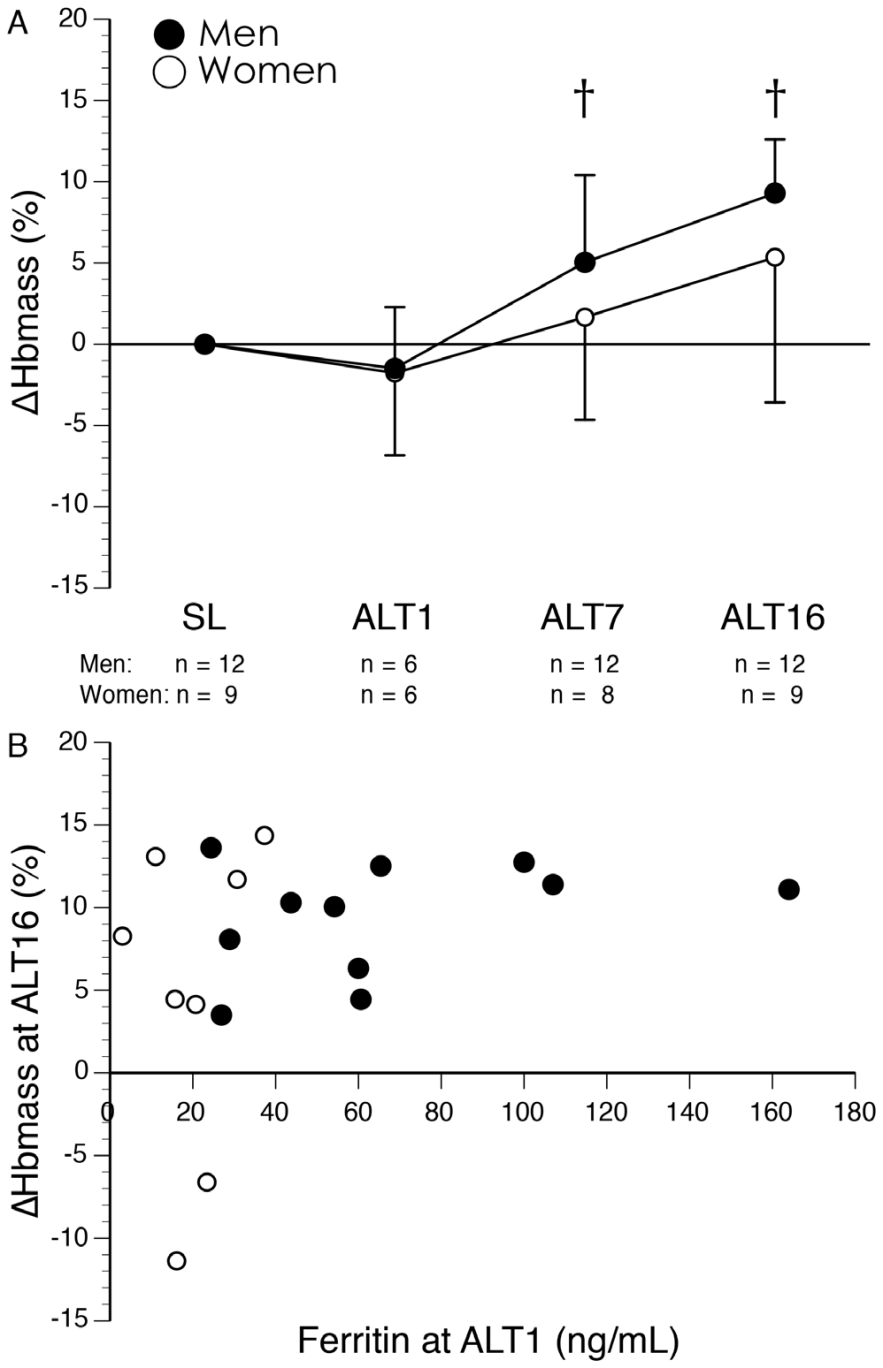
Hemoglobin mass in men and women during 16 days high altitude acclimatization. A) Time course of changes in absolute Hbmass. Data are presented as mean ± SD, with the number of men and women tested at each time indicated below the x-axis. †Significantly different from sea level (p<0.05; main effect of time). The percent changes were not significantly different between men and women (p>0.05). B) Relationship between serum ferritin level upon arrival at high altitude and the percent change in absolute Hbmass following 16 days at high altitude. Two subjects had missing ferritin data at ALT1 and their Hbmass data were excluded from this graph. There was no correlation between initial ferritin level upon arrival at altitude and the percent change in absolute Hbmass during high altitude acclimatization (r = 0.33; n = 19; p = 0.16).

Table 3 presents data on hematological parameters at SL, ALT7, and ALT16. Compared to SL, absolute Hbmass was increased at ALT7 (+3.7±5.8%; n = 20; p<0.01) and ALT16 (+7.6±6.6%; n = 21; p<0.001), with the gain larger at ALT16 compared to ALT7. The increase in absolute Hbmass was larger in men compared to women at ALT16. Relative Hbmass was increased compared to SL at ALT7 and ALT16; relative Hbmass was greater at ALT16 compared to ALT7 and the increases were greater in men than women at both ALT7 and ALT16. Due to the lower absolute and relative Hbmass levels in women compared to men, we also examined the percent change in absolute Hbmass from SL and found no significant difference between men and women (Figure 1A). At ALT16, Hbmass was elevated compared to SL in all 12 men and 7 out of 9 women. There was no significant correlation between ferritin level upon initial exposure to altitude and the percent change in absolute Hbmass at ALT16 (Figure 1B).

**Table 3:** Hematological adaptations during 16 days high altitude acclimatization in healthy men and women.

		Time			Significant Effects		
		SL	ALT7	ALT16	Sex	Time	Interaction
**Hbmass (g)**					**M>W**	**ALT7>SL; ALT16>SL; ALT16>ALT7**	**ΔM>ΔW ALT16**
	**M**	905±95 (12)	950±110 (12)	989±110 (12)			
	**W**	559±62 (9)	567±76 (8)	590±91 (9)			
**Rel Hbmass (g kg^−1^)**					**M>W**	**ALT7>SL; ALT16>SL; ALT16>ALT7**	**ΔM>ΔW ALT7; ΔM>ΔW ALT16**
	**M**	11.9±1.4 (12)	13.0±1.7 (12)	13.8±1.6 (12)			
	**W**	9.1±0.8 (9)	9.2±1.0 (8)	9.7±1.2 (9)			
**v[Hb] (g dL^−1^)**					**M>W**	**ALT7>SL; ALT16>SL**	**ΔM>ΔW ALT16**
	**M**	14.6±0.6 (12)	16.2±1.1 (12)	16.9±0.6 (12)			
	**W**	12.4±0.8 (9)	13.7±1.1 (8)	13.4±1.0 (8)			
**vHct (%)**					**M>W**	**ALT7>SL; ALT16>SL**	**ΔM>ΔW ALT16**
	**M**	44.2±1.4 (12)	48.5±3.0 (12)	50.6±2.2 (12)			
	**W**	39.1±2.3 (9)	42.4±2.8 (8)	42.1±3.3 (8)			
**BV (ml)**					**M>W**	**ALT7<SL; ALT16<SL**	
	**M**	6813±667 (12)	6434±687 (12)	6420±588 (12)			
	**W**	4974±565 (9)	4592±730 (7)	4821±756 (8)			
**Rel BV (ml kg^−1^)**					**M>W**	**ALT16>ALT7**	
	**M**	89.9±9.3 (12)	88.0±8.9 (12)	89.5±8.0 (12)			
	**W**	80.9±7.1 (9)	74.2±7.8 (7)	78.7±9.8 (8)			
**PV (ml)**					**M>W**	**ALT7<SL; ALT16<SL**	**ΔM>ΔW ALT16**
	**M**	4190±412 (12)	3713±473 (12)	3580±304 (12)			
	**W**	3287±417 (9)	2921±502 (7)	3047±499 (8)			
**Rel PV (ml kg^−1^)**						**ALT7<SL; ALT16<SL**	
	**M**	55.3±5.5 (12)	50.7±5.5 (12)	49.9±3.8 (12)			
	**W**	53.4±5.6 (9)	47.2±5.5 (7)	49.8±6.9 (8)			
**RCV (ml)**					**M>W**	**ALT7>SL; ALT16>SL; ALT16>ALT7**	
	**M**	2623±279 (12)	2721±298 (12)	2839±331 (12)			
	**W**	1687±188 (9)	1671±247 (7)	1773±300 (8)			
**Rel RCV (ml kg^−1^)**					**M>W**	**ALT7>SL; ALT16>SL; ALT16>ALT7**	**ΔM>ΔW ALT7; ΔM>ΔW ALT16**
	**M**	34.6±4.0 (12)	37.3±4.6 (12)	39.6±4.8 (12)			
	**W**	27.4±2.3 (9)	27.1±2.8 (7)	28.9±4.0 (8)			

Data are presented as mean ± SD with the number of subjects indicated in parentheses. Linear mixed model statistical analyses were performed to examine the effects of sex, time (with SL as the reference) and a sex × time interaction. Paired t-tests were performed to compare ALT7 with ALT16. Effects were accepted as significant when p≤0.05.

v[Hb] and vHct were increased at ALT7 and ALT16 compared to SL, with no significant differences between ALT7 and ALT16. Men had larger increases in v[Hb] and vHct at ALT16 compared to women. Absolute and relative PV were reduced at ALT7 and ALT16 compared to SL, with no significant difference between ALT7 and ALT16. The reduction in absolute PV at ALT16 was greater in men compared to women, but no significant difference was detected in the change in relative PV. Absolute BV was reduced at ALT7 and ALT16 compared to SL, with no significant difference between ALT7 and ALT16 or in the change in BV between men and women. Relative BV was not significantly different from SL at ALT7 or ALT16, but there was a trend (p = 0.057) for women to have a greater reduction in relative BV compared to men at ALT7. Relative BV was greater at ALT16 compared to ALT7. Changes in absolute and relative RCV mirrored changes in Hbmass.

### Hematological adaptations following descent to low altitude

**Figure 2:**
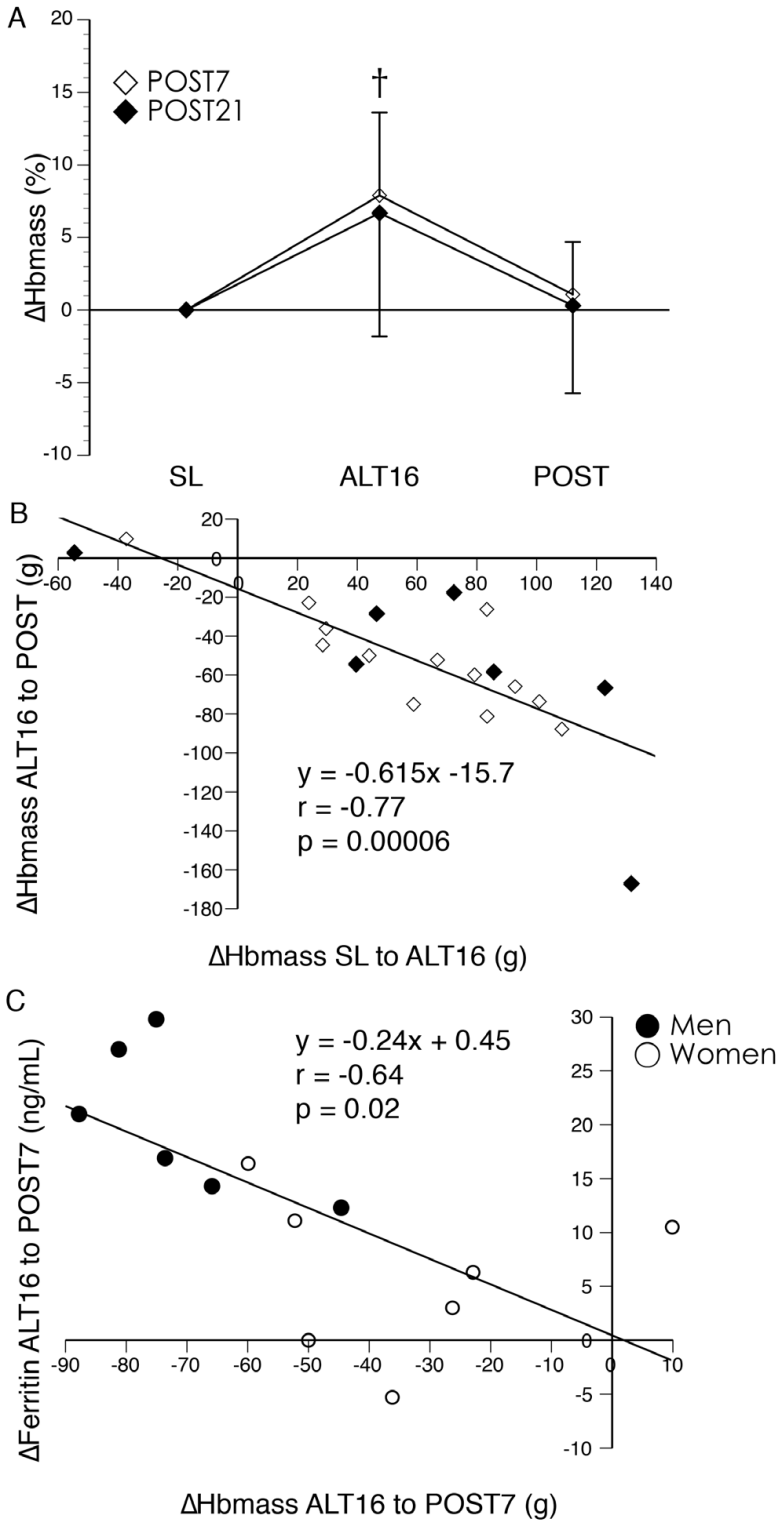
Change in hemoglobin mass following descent from high altitude to low altitude. Subjects were tested at high altitude at the end of a 16 day acclimatization period and upon return to high altitude after descent to low altitude (1525 m) for either 7 (POST7; n = 13) or 21 days (POST21; n = 7). Data are presented as mean ± SD. A) Changes in Hbmass. † Significantly different from POST (p<0.05; main effect of time). There were no significant differences between the POST7 and POST21 groups or between POST and SL (p>0.05). B) Relationship between changes in Hbmass following 16 days high altitude acclimatization and changes in Hbmass following descent to low altitude. C) Relationship between changes in Hbmass and changes in serum ferritin following descent to low altitude for 7 days.

Table 4 presents hematological parameters for subjects with complete measurements at SL, ALT16, and POST7/POST21. For all hematological parameters, there were no significant differences in responses between POST7 and POST21 groups or any significant group × time interactions. Absolute (−6.0±3.7%) and relative (−6.8±4.3%) Hbmass declined following high altitude descent–absolute Hbmass at the POST7/POST21 measurement was not significantly different from SL (+0.8±4.5%), but relative Hbmass was slightly elevated compared to SL (+3.2±5.5%). Figure 2A shows the percent changes in absolute Hbmass from SL at ALT16 and POST7/POST21. A similar pattern was observed for RCV, with absolute and relative RCV values reduced at POST7/POST21 compared to ALT16. Absolute and relative PV were increased at POST7/POST21 compared to ALT16 and not significantly different from SL. Absolute and relative BV at POST7/POST21 were not significantly different from ALT16 or SL. v[Hb] and vHct were reduced at POST7/POST21 compared to ALT16 and not significantly different compared to SL.

**Table 4:** Hematological adaptations following descent from high altitude to low altitude.

		Time			Significant Effects
		SL	ALT16	POST	Time
**Hbmass (g)**					**POST<ALT16**
	**POST7**	726±172 (6,7)	785±194 (6,7)	734±173 (6,7)	
	**POST21**	802±245 (5,2)	865±296 (5,2)	810±266 (5,2)	
**Rel Hbmass (g kg^−1^)**					**POST<ALT16; POST>SL**
	**POST7**	10.2±1.4 (6,7)	11.3±1.9 (6,7)	10.5±1.6 (6,7)	
	**POST21**	11.7±2.4 (5,2)	13.3±3.3 (5,2)	12.3±2.9 (5,2)	
**v[Hb] (g dL^−1^)**					**POST<ALT16**
	**POST7**	13.6±1.2 (5,4)	15.4±1.6 (5,4)	13.8±1.5 (5,4)	
	**POST21**	14.2±1.1 (5,2)	16.0±2.1 (5,2)	14.7±1.8 (5,2)	
**vHct (%)**					**POST<ALT16**
	**POST7**	42.3±2.9 (5,4)	47.0±4.3 (5,4)	42.0±4.1 (5,4)	
	**POST21**	43.1±2.9 (5,2)	48.5±6.1 (5,2)	44.4±4.1 (5,2)	
**BV (ml)**					
	**POST7**	6017±907 (4,4)	5694±926 (4,4)	5965±825 (4,4)	
	**POST21**	6125±1569 (5,2)	5820±1476 (5,2)	5914±1445 (5,2)	
**Rel BV (ml kg^−1^)**					
	**POST7**	83.6±5.2 (4,4)	81.4±7.1 (4,4)	85.0±6.0 (4,4)	
	**POST21**	90.1±13.2 (5,2)	90.2±12.5 (5,2)	90.9±12.0 (5,2)	
**PV (ml)**					**POST>ALT16**
	**POST7**	3813±435 (4,4)	3363±403 (4,4)	3775±423 (4,4)	
	**POST21**	3799±885 (5,2)	3300±664 (5,2)	3587±749 (5,2)	
**Rel PV (ml kg^−1^)**					**POST>ALT16**
	**POST7**	53.2±3.1 (4,4)	48.3±3.0 (4,4)	54.0±4.4 (4,4)	
	**POST21**	56.1±6.9 (5,2)	51.5±4.0 (5,2)	55.4±5.3 (5,2)	
**RCV (ml)**					**POST<ALT16**
	**POST7**	2204±485 (4,4)	2331±556 (4,4)	2190±480 (4,4)	
	**POST21**	2326±695 (5,2)	2520±847 (5,2)	2326±711 (5,2)	
**Rel RCV (ml kg^−1^)**					**POST<ALT16**
	**POST7**	30.4±3.6 (4,4)	33.2±5.3 (4,4)	31.0±4.2 (4,4)	
	**POST21**	34.0±6.7 (5,2)	38.7±9.4 (5,2)	35.5±7.3 (5,2)	

Data are presented as mean ± SD with the number of subjects (M,W) indicated in parentheses. Subjects were studied at sea level, at 5260 m after 16 days high altitude acclimatization, and upon initial return to 5260 m after descent to 1525 m for 7 (POST7) or 21 (POST21) days. Linear mixed model statistical analyses were performed to examine the effects of time (with POST as the reference), group (POST7 versus POST21) and a time × group interaction. Effects were accepted as significant when p≤0.05. There were no significant effects of group or any significant group × time interactions (all p>0.05).

The gain in Hbmass from SL to ALT16 was correlated with the reduction in Hbmass from ALT16 to POST7/POST21 (Figure 2B; r = −0.77; n = 20; p = 0.00006). The reduction in Hbmass from ALT16 to POST7 was correlated with an increase in serum ferritin (Figure 2C; r = −0.64; n = 13; p = 0.02).

## Discussion

This study provides the first data on early Hbmass alterations in healthy humans with ascent to and descent from altitudes greater than 5000 m. We found an increase in Hbmass at ALT7 and a further augmentation by ALT16. However, the altitude-induced gain in Hbmass was remarkably short-lived, as descent to low altitude resulted in a reduction in Hbmass to baseline values within 7 days. The correlation between the loss in Hbmass and increase in serum ferritin following descent to low altitude suggests that this rapid reduction in Hbmass was mediated by increased red blood cell destruction. Overall, this study demonstrates the capacity for rapid alterations in Hbmass with ascent to and descent from high altitude and suggests the need to further examine mechanisms of erythropoietic adaptations to severe hypoxia.

### Increase in Hbmass during high altitude acclimatization

The veracity of our finding of swift alterations in Hbmass is predicated on the validity and sensitivity of our methodological approach for measuring Hbmass. We have several reasons to believe our measurements were robust and that our findings were not the result of analytical error or artifact. First, CO rebreathing methods have been shown to have low measurement error compared to other methodological approaches for assessing the red cell compartment [39] and we achieved a measurement error of 1.5% from duplicate baseline tests in the present study. At ALT7 and ALT16, the mean increases in Hbmass we observed were 2–5 times greater than our measurement error. Second, we performed quality-control analysis for ΔHbCO% both at SL and high altitude and found near-identical results [34]. Third, the ALT7 and ALT16 measures, at which Hbmass was elevated compared to SL, took place at the same location and with the same equipment and personnel as the POST7 and POST21 measures, at which Hbmass had returned to SL values following a 7 or 21 day de-acclimatization period. Therefore, a spurious inflation of Hbmass only at ALT7 and ALT16 seems unlikely. Taken together, we are confident that our findings reflect true physiological alterations in Hbmass with early ascent to and descent from high altitude and were not caused by normal biological variation or analytical error.

**Figure 3:**
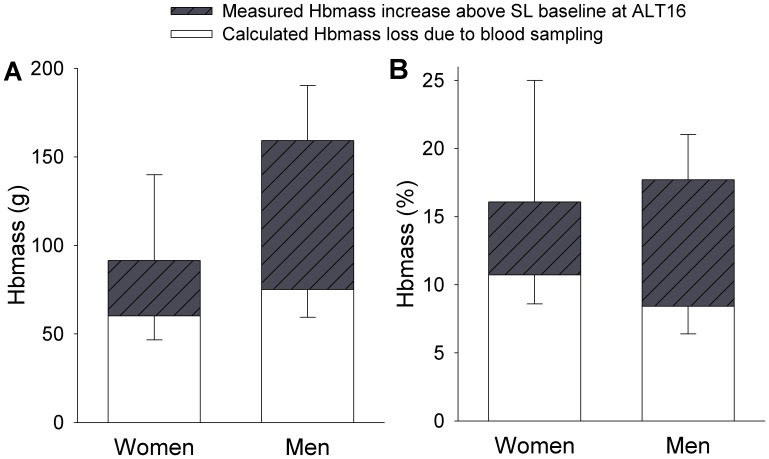
Estimate of the increase in hemoglobin mass produced during 16 days high altitude acclimatization determined from the measured Hbmass increase above sea level baseline plus the calculated Hbmass loss due to blood sampling. Panel A represents the absolute increase in Hbmass (g) produced. Panel B represents the percent increase in Hbmass produced. Data are presented as mean ± SD. Upward SD bars represent the SD of the increase in Hbmass measured above baseline and the downward SD bars represent the SD of calculated Hbmass loss due to blood sampling.

The speed of change in Hbmass observed in our study conflicts with the classical dogma that increases in Hbmass during high altitude acclimatization take at least 3–4 weeks to develop [2], [3]. Recent studies at moderate altitude (2000–3600 m) suggested that erythropoietic adaptations may be swifter than previously thought [13], [14], [15], [17], but the speed and magnitude of increase in Hbmass we observed at ALT7 and ALT16 exceed previous findings. The magnitude of change we observed is particularly striking given that some Hbmass was lost due to sampling (estimated loss of 29±12 g at ALT1, 10±4 g at ALT7, and 30±4 g at ALT16). We report the Hbmass values that we measured without adjusting for the blood lost due to sampling–this underestimates the total increase in the amount of hemoglobin produced at high altitude (estimate presented in Figure 3), which includes the Hbmass change we measured above our SL baseline values plus the Hbmass lost due to blood sampling on ALT1, ALT7 and ALT16 combined (calculated as 69±16 g; n = 21). Our findings raise the intriguing question of what mechanism enables this rapid and robust increase in Hbmass in response to severe hypoxia.

The regulation of red cell production is known to be largely influenced by EPO [42]. EPO peaks within the first 2–3 days of altitude exposure before beginning to fall towards baseline [40], [41] and it has been suggested that the rapid return of EPO to baseline levels with continued altitude exposure should reduce the magnitude of the erythropoeitic stimulus compared to exogenous EPO treatment [2]. However, single measurements of circulating EPO do not adequately reflect the complex kinetics of EPO secretion over days and weeks, and it is unclear if the fall in circulating EPO with continued altitude exposure results from a decrease in EPO expression or is related to an increased rate of clearance from circulation [42]. It is noteworthy that much of the RCV expansion in lowlanders ascending to 4540 m for a 12-month period occurred after 1–2 months [7], a time at which EPO would be expected to have returned to baseline [40], [41]. Importantly, our finding of an increase in Hbmass within 7 days of ascent to high altitude is in stark contrast to studies involving exogenous EPO administration, where Hbmass has been consistently reported to remain unchanged within the first 12 days of treatment [4], [5], [6] despite continuous elevation of circulating EPO above baseline [4]. In comparing high altitude ascent with EPO treatment, it is important to consider that the distinct stimuli of high altitude residence *versus* pharmacological EPO administration are markedly different. Whereas the elevation in EPO with severe hypoxia is secondary to hypoxia-inducible factor (HIF) signaling [43], the provision of exogenous EPO bypasses broad HIF activation. Differences between these conditions are reflected in divergent responses in other pathways affected by HIF signaling that influence erythopoietic adaptations such as iron mobilization [4], [43], [44]. Ultimately, we cannot provide mechanistic data from our study explaining the swifter increase in Hbmass in our subjects compared to previous studies at lower elevations or involving exogenous EPO administration, and we are not suggesting that EPO is not a key player in augmenting erythropoiesis in response to severe hypoxia. Rather, the more rapid increase in Hbmass in severe hypoxia compared to exogenous EPO treatment suggests that mechanisms in addition to augmentation of EPO may play an important role in the rapid erythropoietic response.

To our knowledge, we are the first to compare changes in Hbmass/RCV in men and women at identical time points and under similar experimental conditions at altitudes greater than 4000 m. We found that the percent increase in absolute Hbmass following 16 days at high altitude was not significantly different between men and women. Some previous cross-sectional studies of moderate altitude residents have suggested that erythropoietic responses may be lower in females compared to males [45], [46], and it has been suggested that the ventilatory-stimulating effects of the female sex hormones play a key role [47]. However, Reeves et al. found no effect of menstrual phase on ventilatory or erythropoietic adaptations in healthy women acclimatizing to 4300 m despite large differences in sex hormone levels between subjects in the luteal versus follicular phases [12]. Arterial oxygen pressure and saturation did not differ between men and women at ALT1 and therefore the impact of ventilatory effects on potential sex differences in erythropoietic responses to high altitude would be minimal in our study.

Our finding that the percent change in absolute Hbmass did not differ between men and women is particularly striking given the low ferritin levels of our female subjects upon arrival at high altitude. Although subjects with low ferritin during baseline testing were directed to take oral iron supplements up until departure for high altitude, several women arrived at high altitude with low ferritin levels, and based on previous work at moderate altitude [48], one might expect that the low iron stores would prevent an increase in Hbmass. In contrast, most (7 out of 9) of the women increased Hbmass in response to high altitude exposure. However, while all 12 men had increases in Hbmass following 16 days high altitude acclimatization, 2 women failed to increase Hbmass; indeed, these 2 women had reductions in Hbmass that were similar to the calculated amount of Hbmass withdrawn from these subjects for blood sampling at altitude. We examined the EPO response of these 2 individual subjects and found that their increases in EPO with high altitude exposure were above the group median at ALT1, ALT7, and ALT16, suggesting that the failure to increase Hbmass was not caused by a lack of EPO upregulation. As can be observed in Figure 1B, these 2 women were not distinguished by particularly low iron stores upon arrival at high altitude. Indeed, our individual data demonstrate the capability to increase Hbmass despite low ferritin levels upon initial arrival at high altitude. The subject with the lowest ferritin (3 ng mL^−1^) upon initial high altitude exposure had a relatively large (8.3%) increase in Hbmass.

It might be questioned whether the level of storage iron indicated by these low ferritin values would be sufficient to enable a large increase in Hbmass. However, previous work has demonstrated increases in intestinal iron absorption at high altitude [7], [49], [50] and it is possible that dietary iron intake (not measured in the current study) provided sufficient iron for increasing hemoglobin production. Additionally, recent data suggest that a decrease in skeletal muscle iron content during the first week at high altitude may increase iron available for erythropoiesis [44]. Admittedly, because we did not measure dietary iron intake or iron-related proteins in skeletal muscle, the role of these mechanisms in allowing increased erythropoiesis in our subjects with low ferritin is purely speculative. However, these other studies highlight the complexity of iron homeostasis at high altitude and suggest that the iron required for increasing erythropoiesis may have been obtained from increased intestinal absorption or mobilization of iron from skeletal muscle stores. Further studies involving Hbmass assessments coupled with more comprehensive assessments of iron homeostasis are needed to more robustly determine the relationship between iron availability and erythropoiesis at high altitude. Our data suggest that low initial iron stores do not requisitely prevent high altitude-induced erythropoiesis; however, we stress that we cannot determine from our data whether low iron stores limited the magnitude of increase in Hbmass in some subjects.

### Decrease in Hbmass following descent from high altitude to low altitude

We found that the Hbmass gained during high altitude acclimatization was quickly lost following descent to 1525 m. Hbmass had returned to SL baseline in our subjects who descended to low altitude for 7 days, and there was no further decrement in the group who descended for 21 days. To our knowledge, we are the first to report a complete loss of altitude-induced Hbmass adaptation within 7 days; the speed of this de-acclimatization response contrasts with previous studies, in which Hbmass has been reported to remain elevated above baseline for multiple weeks following descent to SL [14], [15], [17], [51]. Although a rapid loss in RCV following high altitude descent has been previously reported by Rice et al. [21], there are several aspects of our study that make our findings unique. We studied lowlanders following just 16 days of high altitude acclimatization whereas Rice et al. [21] studied polycythemic high altitude natives. This was reflected by dramatic differences in the degree of polycythemia obtained at high altitude (mean [Hb] of 23.4 g dL^−1^ in the high altitude natives versus 15.5 g dL^−1^ at ALT16 in our subjects). Indeed, the majority of subjects in the study of Rice et al. [21] met the criteria for excessive erythrocytosis ([Hb]≥21 in men or ≥19 in women; [52]) whereas none of our subjects came within 2 g dL^−1^ of this criterion at ALT16. Therefore, our results show that the development of excessive polycythemia is not required for high altitude descent to induce a rapid loss in Hbmass.

Based on the kinetics of red blood cell turnover (∼0.83% of circulating cells destroyed per day [53]) and the delayed influence of changes in EPO on red blood cell production [21], [42], the large reduction in Hbmass we observed within 7 days is unlikely to be explained by a reduction in red blood cell production. The correlation between the loss in Hbmass and increase in serum ferritin from ALT16 to POST7 suggests an increase in red blood cell destruction, as the iron contained in destroyed red blood cells is transferred to iron stores [21]. It is possible that neocytolysis, the selective destruction of a population of young red blood cells [54], [55], [56], may have been the mechanism of this rapid loss in Hbmass. However, the strength of the evidence for neocytolysis has recently been questioned [57]. We did not measure markers of red blood cell production or examine red blood cell age distributions during high altitude acclimatization and de-acclimatization and therefore cannot provide direct evidence in support of, or against a role for, neocytolysis.

Our finding of a rapid loss in Hbmass following descent from high altitude contrasts with patterns observed following cessation of exogenous EPO administration [5], [6], [20], despite the fact that these studies induced similar or larger elevations in Hbmass and [Hb]/Hct compared to our observations. Although it was hypothesized that cessation of exogenous EPO therapy would induce neocytolysis and lead to a rapid reduction in Hbmass [21], [56], recent studies provide compelling evidence that this is not the case, with Hbmass/RCV consistently reported to remain unchanged for 2 weeks following treatment cessation before beginning to fall gradually back to baseline [5], [6], [20]. The stimulus for increased red blood cell production with high altitude acclimatization is hypobaric hypoxia, whereas EPO treatment elevates Hbmass in the absence of systemic hypoxia. Although this suggests that the production of red blood cells under conditions of hypoxia may influence the retention of Hbmass adaptations, it is important to note that rapid reductions in Hbmass have also been reported with spaceflight [54] and dehydration-induced rapid weight loss [58] and neither of these situations involve systemic hypoxia. Further work is required to clearly establish the mechanism(s) of rapid loss of Hbmass in healthy humans.

What are the implications of the rapid loss in Hbmass following descent to low altitude on acclimatization status upon return to high altitude? The reduction in oxygen carrying capacity might be expected to impair submaximal endurance performance upon return to 5260 m; however, despite large reductions in absolute and relative Hbmass from ALT16 to POST7, the improvement in 3.2 km run time-trial performance from ALT1 to ALT16 with acclimatization was fully maintained at POST7 [22]. This calls into question the importance of the altitude-induced Hbmass adaptation for submaximal endurance performance at high altitude. Previous studies examining the effects of artificial Hbmass alterations with erythrocyte infusion [59], [60], recombinant EPO treatment [61], and isovolemic hemodilution [62] have failed to observe alterations in maximal oxygen uptake or endurance performance at altitudes greater than 4300 m. Our results extend these findings by showing that the loss in Hbmass accompanying descent to low altitude does not result in impaired submaximal endurance performance upon return to high altitude. However, previous work suggests that there may be a threshold altitude above which alterations in Hbmass have minimal effects on maximal oxygen uptake [61], and we stress that our finding of maintained endurance performance despite significant loss of Hbmass may not apply to performances at less severe altitudes.

### Changes in blood volume compartments during high altitude acclimatization and de-acclimatization

In support of previous high altitude studies (Reviewed in [2]), we observed large reductions in absolute and relative PV at ALT7 and ALT16. However, we did not detect a significant reduction in PV in the first 9–13 hours of initial exposure to 5260 m and the PV measured 9–13 hours after return to 5260 m following descent to low altitude was not different from SL. Some studies have found reductions in PV within the first hours of high altitude exposure [9], [10] but other studies have failed to detect changes within this time window [23], [24]. Differences between studies are likely influenced by several factors including hydration status, exercise prior to PV assessments, acute mountain sickness, and the methodology used to assess PV. PV returned to SL values following descent to low altitude. Previous work has indicated that the recovery of PV following high altitude descent occurs within 2 days and is influenced by changes in fluid-regulating hormones including renin, aldosterone, and vasopressin [26], [27], so our finding of a recovered PV following 1 and 3 weeks at low altitude is not surprising. As expected, changes in RCV paralleled the changes we observed in Hbmass. Compared to SL, relative BV was unchanged by high altitude acclimatization and de-acclimatization, at least at the time points we assessed–relative BV was influenced both by alterations in the absolute sizes of the BV compartments and small changes in body mass [22] during high altitude acclimatization and de-acclimatization. In our subjects, reductions in relative PV during high altitude acclimatization were offset by an augmentation of relative RCV. The opposite response occurred following descent to low altitude, with the diminution in relative RCV offset by an enlargement of relative PV.

### Limitations

There are some potential limitations to the current study that should be considered. The overall experimental design lacked a separate lowlander control group that was studied over time in the absence of altitude exposure. However, Hbmass has been consistently shown to be stable over time in subjects at SL [36], [37] and we took several steps to ensure that the SL and high altitude measurements were comparable, as detailed above. Additionally, because examining responses to acute hypoxia on ALT1 was a key component of the overall AltitudeOmics study design, many steps were taken to minimize the subjects' exposure to hypoxia prior to ALT1 [22]. During the travel period prior to ALT1 (including flight time), subjects spent less than 20 hours exposed to hypoxia equivalent to 2000 m or greater. A recent meta-analysis of changes in Hbmass with hypoxia reported gains in Hbmass of ∼1% per 100 hours spent above 2000 m [17]. Therefore, the effect of hypoxic exposure prior to ALT1 on the Hbmass response is estimated as less than 0.2%, dramatically lower than the increases we observed at ALT7 and ALT16.

As described previously, subjects were unable to maintain their normal physical activity habits at high altitude and some detraining may have occurred during acclimatization, with some fitness restoration during the period spent at low altitude [22]. Eastwood et al. found a 3.1% reduction in Hbmass after 30 days of detraining (∼90% reduction in training volume) in triathletes at SL, but reported unchanged Hbmass at 10 and 20 days following training reduction [63]. A potential interaction between hypoxia and detraining on changes in Hbmass with ascent to high altitude has not been previously examined. There is a very strong cross-sectional relationship between lean body mass and Hbmass at sea level [64] and it could be speculated that the mean loss of ∼1.5 kg lean body mass between ALT1 and ALT16 [22] may have reduced the erythropoietic stimulus. However, data examining a potential interaction between changes in lean body mass and Hbmass during altitude sojourn are currently lacking. Next, although subjects with low ferritin prior to baseline testing were directed to take oral iron supplements, supplementation was not directly monitored and the efficacy of supplementation in increasing serum ferritin was not determined prior to arrival at high altitude. Some subjects arrived at high altitude with low ferritin levels and it is possible that this may have limited the increase in Hbmass. However, as noted above, several subjects had robust increases in Hbmass despite low ferritin levels upon arrival.

Finally, the potential influence of blood loss due to sampling should be considered. Blood loss due to sampling occurs in many studies but its potential influence on hematological and other physiological outcomes is often ignored. The amount of blood removed due to sampling at ALT1 is of a magnitude that may induce a small EPO response at SL [65]. It is important to consider that it takes ∼5 weeks to recover Hbmass lost from a 550 mL blood donation at SL [66], whereas our subjects were able to increase Hbmass above SL baseline within 7 days despite the loss of blood due to sampling. While we cannot rule out a potential interaction between blood loss due to sampling and the hypoxic stimulus on the magnitude of the erythropoietic response, it is clear that the hypoxic stimulus drives the rapid gain in Hbmass observed at 5260 m.

## Conclusions

We documented the early time course of Hbmass adaptations at 5260 m and found rapid increases following just 7 and 16 days of high altitude acclimatization. The altitude-induced gain in Hbmass was remarkably short-lived, as descent to low altitude resulted in a dramatic loss in Hbmass within 7 days. The loss in Hbmass was correlated with an increase in serum ferritin, suggesting an increase in red blood cell destruction. Overall, this study demonstrates the capacity for rapid alterations in Hbmass with high altitude acclimatization and de-acclimatization in healthy men and women and suggests the need to further examine mechanisms of erythropoietic adaptations to severe hypoxia.

## Supporting Information

Table S1 **Individual hemoglobin mass data at SL, ALT1, ALT7, ALT16, POST7, and POST21 and serum ferritin data at SL, ALT1, ALT16, POST7, and POST21.**
(PDF)
