# Supplementary material for: AltitudeOmics: Rapid Hemoglobin Mass Alterations with Early Acclimatization to and De-Acclimatization from 5260 m in Healthy Humans
Source: PLoS One. 2014 Oct 1;9(10):e108788. doi: 10.1371/journal.pone.0108788 (PMC4182755; doi:10.1371/journal.pone.0108788)
Supplement: Table S1 — Individual hemoglobin mass data at SL, ALT1, ALT7, ALT16, POST7, and POST21 and serum ferritin data at SL, ALT1, ALT16, POST7, and POST21. (PDF) [file pone.0108788.s001.pdf]

	Hbmass (g)						Serum Ferritin (ng/mL)				
**ID**	**SL**	**ALT1**	**ALT7**	**ALT16**	**POST7**	**POST21**	**SL**	**ALT1**	**ALT16**	**POST7**	**POST21**
**001**	966		966	1098		931	26	24	6		21
**002**	891		917	930		876	34	61	17		47
**003**	459		511	506		477	27		8		72
**004**	969		1139	1041		1023	53		85		
**005**	480	443	441	425		428	34	16	3		5
**006**	772		809	858		799	117	164	64		89
**007**	1077		1114	1200		1133	84	107	21		172
**010**	563	516	540	525	535		24	23	6	17	
**011**	664	657	670	693	657		25	16	10	4	
**012**	827	837	894	912			41	44	13	22	
**013**	923	873	1014	1016	950		83	54	10	25	
**014**	1032	993	1036	1115	1034		34	29	11	38	
**015**	605	629	625	684	625		11	11	4	20	
**017**	580	592	627	663	637		39	37	12	15	
**018**	806	786	804	907	833		87	65	18	35	
**019**	532	534	522	576	526		10	3	0	0	
**020**	575		597	599	576		30	21	17	23	
**021**	813	786	832	841	796		70	27	21	33	
**022**	930		943	989	914		40	60	17	47	
**023**	851	891	934	959	871		90	100	26	47	
**025**	571			637	585		61	31	10	21	
